# DOSim: An R package for similarity between diseases based on Disease Ontology

**DOI:** 10.1186/1471-2105-12-266

**Published:** 2011-06-29

**Authors:** Jiang Li, Binsheng Gong, Xi Chen, Tao Liu, Chao Wu, Fan Zhang, Chunquan Li, Xiang Li, Shaoqi Rao, Xia Li

**Affiliations:** 1 College of Bioinformatics Science and Technology, Harbin Medical University, 194 Xuefu Road, Harbin 150081, China

## Abstract

**Background:**

The construction of the Disease Ontology (DO) has helped promote the investigation of diseases and disease risk factors. DO enables researchers to analyse disease similarity by adopting semantic similarity measures, and has expanded our understanding of the relationships between different diseases and to classify them. Simultaneously, similarities between genes can also be analysed by their associations with similar diseases. As a result, disease heterogeneity is better understood and insights into the molecular pathogenesis of similar diseases have been gained. However, bioinformatics tools that provide easy and straight forward ways to use DO to study disease and gene similarity simultaneously are required.

**Results:**

We have developed an R-based software package (DOSim) to compute the similarity between diseases and to measure the similarity between human genes in terms of diseases. DOSim incorporates a DO-based enrichment analysis function that can be used to explore the disease feature of an independent gene set. A multilayered enrichment analysis (GO and KEGG annotation) annotation function that helps users explore the biological meaning implied in a newly detected gene module is also part of the DOSim package. We used the disease similarity application to demonstrate the relationship between 128 different DO cancer terms. The hierarchical clustering of these 128 different cancers showed modular characteristics. In another case study, we used the gene similarity application on 361 obesity-related genes. The results revealed the complex pathogenesis of obesity. In addition, the gene module detection and gene module multilayered annotation functions in DOSim when applied on these 361 obesity-related genes helped extend our understanding of the complex pathogenesis of obesity risk phenotypes and the heterogeneity of obesity-related diseases.

**Conclusions:**

DOSim can be used to detect disease-driven gene modules, and to annotate the modules for functions and pathways. The DOSim package can also be used to visualise DO structure. DOSim can reflect the modular characteristic of disease related genes and promote our understanding of the complex pathogenesis of diseases. DOSim is available on the Comprehensive R Archive Network (CRAN) or http://bioinfo.hrbmu.edu.cn/dosim.

## Background

The past several decades have seen a number of methods applied to the computation of similarities between diseases [1-4]. The early work used clinical phenotypes or diagnosed information. For example, Kalaria [1] ascertained similarities between Alzheimer's disease and vascular dementia by studying the similarities between disease symptoms and pathological result. More recently, with the availability of large-scale knowledge bases such as the Online Mendelian Inheritance in Man (OMIM) [5] and the Genetic Association Database (GAD) [6], scientists are able to explore the genetic similarity between diseases. In 2009, Liu et al. [7] revealed similarities between diseases by combining both genetic (data from GAD [6]) and environmental (data from Medical Subject Headings, MeSH [8]) factors and, by mining for disease etiologies, created a new concept named the "etiome". Zhang and his colleagues [9] used a text-based method to build up a human disease phenotype network in which a disease was represented by a feature vector and the similarities between two diseases were calculated as the cosine of the angle between their corresponding feature vectors. However, little work has been done to apply semantic similarity measures between diseases using ontology, another way to analyze relationship between diseases.

Understanding similarities between genes has a significant role to play in disease research. One hypothesis states that genes associated with similar diseases have similar functions; the greater the gene similarity the higher the probability that the genes are associated with similar similarity. However, current methods to determine gene similarity rely on sequence similarity, gene expression profiles, Gene Ontology (GO) [10] annotations or PubMed abstracts, all of which are derived from normal or partially abnormal conditions and it secludes gene similarity from disease similarity. Thus, a process to determine the similarities between genes in terms of diseases and to map gene similarities to disease similarities would help us better understand the mechanism of complex diseases.

**Figure 1:**
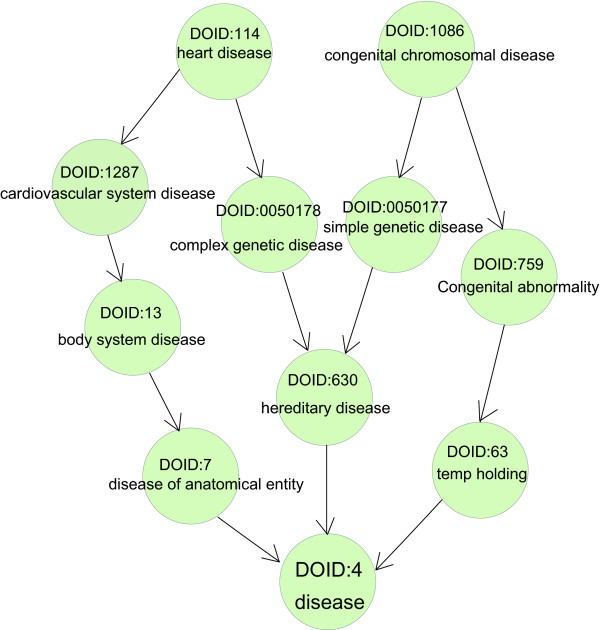
**Example of a sub-DO DAG**. Example of a sub-DO DAG starting with leaves of DOID:114 (heart disease) and DOID:1086 (congenital chromosomal disease).

The Disease Ontology (DO) aims to provide an open source ontology for the integration of biomedical data that is associated with human disease [11]. The terms in DO are disease names or disease-related concepts and are organised in a directed acyclic graph (DAG) (Figure 1). Two linked diseases in DO are in an 'is-a' relationship, which means one disease is a subtype of the other linked disease. And the lower a disease is in the DO hierarchy, the more specific the disease term is. A recent work by Osborne and his colleagues [12] in which they used DO to annotate the human genome, further advanced the application of DO. Recently, a simplified vocabulary list, Disease Ontology Lite (DOLite), was shown to give more interpretable results than DO in gene-disease association tests. DOLite has been used in FunDO (Functional Disease Ontology) [13], one of the few bioinformatics tools based on DO that aims to explore disease information implied in the gene set. This work makes it possible to study disease similarity and gene similarity simultaneously in DO using the annotated human genome. Thus, we developed DOSim, an R package for the computation of DO-based similarity between diseases in an ontology sense. DOSim was developed on DO, subversion 926; the DO term annotations of the human genes in DOSim were taken from the study of Osborne et al. [12]. A total of 4054 genes have been assigned DO term annotations. Compared with FunDO, DOSim divides functions into three categories: (i) measuring the similarity between diseases (DO terms), (ii) measuring the similarity between human genes in terms of diseases, (iii) other utilities for conducting DO enrichment analysis (similar to FunDO), detecting and annotating DO-directed gene modules, and describing and visualizing DO structures and terms.

## Implementation

### Measuring the similarity between diseases

Terms in DO include disease names and disease-related concepts. Exploring the similarity between them can help us to understand the relatedness between diseases. The past few years have seen an increase in the number of different measures used for the calculation of semantic similarity. Based on the semantic similarity measures in the application of biomedical ontologies reviewed by Pesquita etc al. [14], for general applicability, in DOSim we implemented ten representative semantic similarity measures, which are Resnik measure [15], Lin measure [16], Jiang and Conrath measure (JC) [17], Relevance measure (Rel) [18], Graph Information Content measure (GIC) [19], Information Coefficient similarity measure (simIC) [20], Wang measure [21], modified Resnik measure (CoutoResnik) [22], modified Lin measure (CoutoLin) [22], and modified Jiang and Conrath measure (CoutoJC) [22]. Except for the Wang measure that uses a hybrid measure, the other nine measures are based on information content (IC).

The IC of a term/disease *t *in the DO database gives a measure of how specific and informative a term/disease is, and is defined as *IC*(*t*) = -log *p*(*t*), where *p*(*t*) is the number of genes annotated to the term *t *and its descendants divided by the total number of genes annotated to DO. When characterizing the shared IC between two terms, two concepts, most informative common ancestor (MICA) and disjunctive common ancestor (DCA), are widely used[22]. The MICA of two terms *t*_*1 *_and *t*_*2 *_is the one that possesses the maximum IC among all the common ancestor terms of the two terms. And the DCAs of two terms *t*_*1 *_and *t*_*2 *_are the MICA of disjunctive ancestors of the two terms, which can be defined as follows:(1)

where disjunctive ancestors of the term *t*, *DisjAnc*(*t*), can be described as that two ancestors *a*_*1 *_and *a*_*2 *_are disjunctive ancestors of the term *t *if there is a path from *a*_*1 *_to *t *not passing through *a*_*2 *_and a path from *a*_*2 *_to *t *not passing through *a*_*1*_. It can be formulated as follows:(2)

Then, the shared information of two terms *t*_*1 *_and *t*_*2*_, *Share*(*t*_*1*_*,t*_*2*_), is defined as the average of the IC of the DCAs, formulated as:(3)

Let *t*_*MICA *_represent the MICA term of two terms *t*_*1 *_and *t*_*2*_, then the nine IC-based similarity measures are calculated as follows:(4)(5)(6)(7)(8)(9)(10)(11)(12)

In the Wang measure, each edge is given a weight according to the types of relationships. For a term *A*, a sub-DAG comprised of the term *A *and all its ancestor terms can be represented as *DAG*_*A *_= (*A,T*_*A*_*,E*_*A*_), where *T*_*A *_is the ancestor term set of term *A *(including *A *itself) and *E*_*A *_is the set of edges connecting to the terms in *DAG*_*A*_. For any term *t *in *DAG*_*A*_, Wang et al. [21] defined the semantic contribution of *t *to *A*, *DA*(*t*), as the product of all the edge weights in the "best" path from term *t *to *A*, where the "best" path is the one that maximises the product (the semantic contribution of the term *A *to itself is set to 1). It can be represented as follow:(13)

where *w*_*e *_is the semantic contribution factor of edge *e *(*e *∈ *E*_*A*_). It is set between 0 and 1 according to the types of relationships, e.g., "is-a" or "part-of". In DO, there is only one type of relationship, defined as "is-a". In DOSim, we set *w*_*e *_to 0.7.

The semantic similarity between two terms *A *and *B *is then calculated as follows:(14)

where *SV*(*A*) (or *SV*(*B*)) is the total semantic contribution of the term *A *(or *B*) in *DAG*_*A *_(or *DAG*_*B*_), which is calculated as:(15)(16)

### Measuring the similarity between human genes in terms of diseases

In the DOSim package, the similarity between two genes based on the similarity of their DO term annotation groups is calculated. Each gene is represented by its set of direct DO term annotations, and semantic similarity is calculated between terms in one set and terms in the other (using one of the measures described above). Some methods consider every pairwise combination of terms for the two sets, while others consider only the best-matching pair for each term. Five different methods are implemented in DOSim; they are the arithmetic maxima and average of pairwise similarity between two groups of DO terms describing the two genes (Max, Mean) [23], the arithmetic maxima and average between similarities for two directional comparisons of the similarity matrix *S *of two genes (funSimMax, funSimAvg) [18], and the best-match average approach (BMA) [21] which considers the contributions from the semantically similar terms that annotated the two genes respectively (Formula 23).

Let *DO*_*1 *_and *DO*_*2 *_be the groups of annotation terms for two genes *g*_*1 *_and *g*_*2*_, and *m *and *n *are the number of terms in *DO*_*1 *_and *DO*_*2 *_respectively. A similarity matrix *S=*[*s*_*ij*_]_*m×n *_contains all pairwise similarity scores of mappings from *DO*_*1 *_to *DO*_*2 *_when you refer to each row and vice verse when you refer to each column. '*rowScore*' and '*columnScore*' of *S *are the averages over the row maxima and the column maxima, which give similarity scores for the comparison of *DO*_*1 *_to *DO*_*2 *_and the comparison of *DO*_*2 *_to *DO*_*1*_, respectively.(17)(18)

Using these definitions, the five similarity methods for the computation of gene similarity between two genes *g*_*1 *_and *g*_*2 *_are defined as follows:(19)(20)(21)(22)(23)

For a set of genes *G *(*g*_*1*_*,g*_*2*_*,...,g*_*n*_) of size *n*, the similarity matrix for these genes is defined as *Sim*=[*Sim*_*ij*_]_*n×n*_, where *Sim*_*ij *_is the similarity between gene *g*_*1 *_and *g*_*j *_derived by any of the five methods defined above.

In DOSim, there are a total of fifty optional semantic similarity measures for genes, which are combinations of the ten semantic similarity measures for term pairs and the five similarity methods mentioned above.

### Other utilities

#### Conducting DO enrichment analysis

In DOSim, DO-based enrichment analysis is implemented to explore the disease feature of an independent gene set, for example, a differentially expressed gene set from a microarray analysis. Significance of the enrichment analysis is assessed by the hypergeometric test and the *p*-value is adjusted by false discovery rate (FDR). For a certain DO term *t *which meets the requirement (see below), if *M *genes are the number of annotated genes in the human genome and *x *genes are the number of annotated genes in the gene set for this term, then to calculate whether the gene set is enriched in DO term the following formula is used:(24)

where, *N *is the total number of human genes in the genome, *k *is the size of the gene set of interest, and  is the number of combinations of the *N *genes taken *k *at a time and is equal to .

Compared with FunDO, which uses a small set of DO terms (DOLite) [13], DOSim selects the DO terms satisfy two criteria for enrichment analysis, aiming at exploring more biological result. The first criterion is that the term should be annotated by at least *n *genes, and the second is that the term should be beneath a depth *m *in the DAG of DO, where *n *and *m *can be set by users when running the DO enrichment analysis.

In the DOSim package, the *DOEnrichment *function carries out the DO enrichment analysis; the input is a list of Entrez gene IDs. The *filter *and *layer *parameters are the two criteria mentioned above that can be used to control the terms to be analysed; so that the term is annotated by at least 'filter size' genes and it is beneath the 'layer' depth in the DAG of DO.

### Detecting and annotating DO-directed gene modules

A gene module is a group of highly correlated genes. In DOSim, gene modules can be detected as follows: after the gene similarity matrix for a gene set is constructed, a hierarchical clustering is performed using the standard R function *hclust *and one of three branch cutting methods is applied (one constant-height cutting and two dynamic branch cutting methods are embed in our package) [24].

The DOSim package incorporates multilayered enrichment analysis (GO and KEGG annotation) to explore the biological meaning of the detected gene modules. The GO annotations are conducted using GOSim [25] and the KEGG annotations are generated using SubpathwayMiner [26]. The input for GO and KEGG annotations is a list of Entrez gene IDs, the mechanism implied in each annotation database is the hypergeometric test, and the outputs for each annotation database are the enriched terms with *p*-values.

### Describing and visualizing DO structures and terms

DO is a collection of terminologies associated with human diseases and the terms in DO are organised in a DAG (Figure 1). DOSim also provides useful utilities to easily visualise the DO structure; thus users need not turn to other tools (e.g., OBO-Edit). Specifically, the hierarchical structures of DO terms can be represented as a *graphNEL *object and the *getDOGraph *function in DOSim can be used to fetch the DO graph with specified DO terms at its leaves. For a certain DO term, DOSim provides a series of functions to extract related terms (e.g., father and child terms.).

## Results

### The effect of different measures on the computation of gene similarity

**Figure 2:**
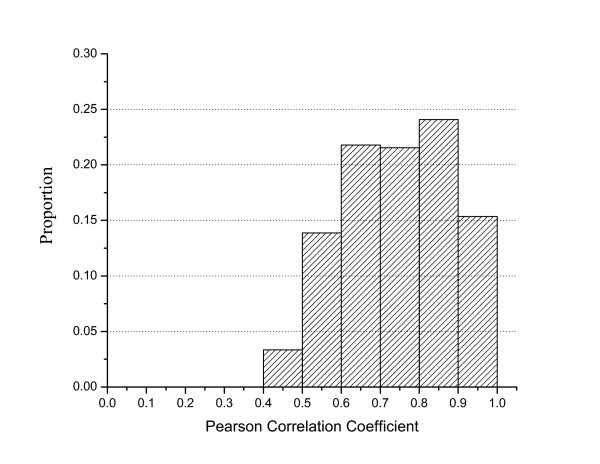
**Distribution of the Pearson correlation coefficient of gene similarity scores between parameter combinations**.

The different similarity measures for both the terms and the genes have their advantages when applied to biomedical ontologies [14]. An important question that we addressed was, do different similarity measures for the same gene pairs produce very different results? We used all the fifty similarity measures implemented in DOSim to calculate the similarities between the 4045 genes that have DO annotations. A Pearson correlation coefficient (PCC) analysis between the gene similarities calculated using the different similarity measures was then carried out to quantify the influence of the similarity measures. The resultant PCC frequency distribution (Figure 2) showed that the gene similarities calculated by the different similarity measures were closely correlated, indicating that the different similarity measures do not much significantly influence the computation of gene similarity.

### Application on disease similarity

We investigated the relationships between different kinds of cancers using disease similarities derived from DOSim. First, 128 cancer disease DO terms were obtained by using "cancer" as the key word to search all DO term names (exclude the DO term, "DOID:162, cancer"). Then, we used the *getTermSim *function to get the pairwise similarities using Wang measure (This is an example here. Users can choose any of the other measures in their applications).

**Figure 3:**
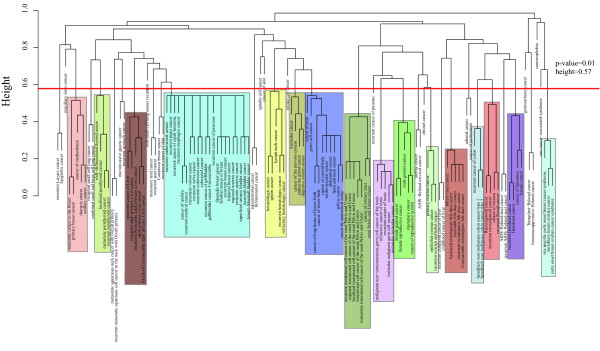
**Hierarchical clustering of 128 cancer terms**. The distance between two diseases is defined to be 1- the Wang's similarity of the two diseases. The tree was constructed using the average method of hierarchical clustering. The red line corresponds to a *p*-value of 0.01. Disease correlations below this line are considered significant. The different colours represent the various categories of significant disease correlations.

Figure 3 is the average linkage hierarchical clustering of the 128 different cancer terms based on the similarities computed by the Wang measure. To assign significance to these associations, we randomly selected 128 diseases from all the diseases covered by DO terms and calculated the similarities among them. This process was repeated 100 times to generate a background distribution. The background distribution value at the 99th percentile was 0.43 (*p*-value = 0.01). Only those disease correlations that passed the *p*-value threshold of 0.01 were selected. Using this criterion we found 800 significant disease-disease similarity relationships. We defined a "module" as a sub-branch in the hierarchical clustering which had at least three diseases and under a height of 0.57 (inverse of similarity). This resulted in 16 modules with sizes ranging from 3 to 22. Generally, many of the expected disease associations that pooled together in one sub-branch were those that we expected; for example, the thyroid-related cancers, well-differentiated thyroid cancer (DOID:3971), localised parathyroid cancer (DOID:1544), metastatic parathyroid cancer (DOID:7149) and recurrent parathyroid cancer (DOID:7150) were all in one module. Many novel and hitherto unknown significant correlations such as the similarity between hematologic cancer (DOID:2531) and spleen cancer (DOID:672) which had a similarity of 0.785 were discovered. The spleen is part of the lymphatic system which can filter the blood and help the body fight infections. Lymphoma is a type of hematologic cancer that develops in the lymphatic system. Malignant lymphoma can occur in various organs, including the spleen [27] and among the causes of isolated splenomegaly, lymphoid malignancies account for a relevant, yet probably underestimated, number of cases [28]. Taking the correlation between hematologic cancer and spleen as an example, such relationships can be easily explored by DOSim.

**Figure 4:**
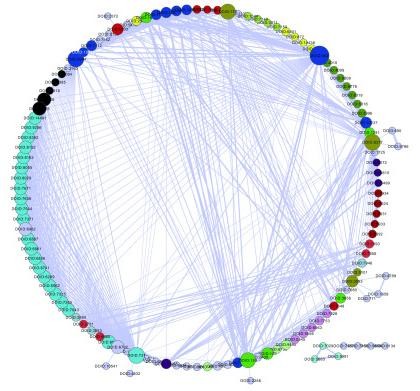
**The network of all the 128 cancer terms**. The colours correspond to the significant disease correlation categories in Figure 3. The nodes coloured in grey are not grouped in Figure 3. The thickness of the edges between two diseases represents the strength of their correlation.

We also created a network representation to display all the 800 significant disease correlations by using the Cytoscape software package [29] (Figure 4). In the network, the nodes were diseases, and the thickness of the edges between two diseases represented their strength of correlation. The network revealed strong correlations between different modules (defined in hierarchical clustering), which helped us to pick additional significant disease associations that were missing in the hierarchical clustering. For example, germ cell cancer (DOID:2994), a member of the module labelled in blue with size 10, correlated with almost every member of the largest module of size 22. This network application demonstrates that, although cancer diseases show modular characteristics, they are also highly correlated with each other. A detailed pairwise similarity matrix between the 128 cancer terms and a list of significant cancer pairs are provided in Additional file 1.

We also constructed the DO graph of these 128 cancers as leaves (Additional file 2), which finally contained 398 disease DO terms. We found that, as expected, diseases in the same module represented hierarchical structure in the DO graph as illustrated in the Figure S1. For example, the module marked brown contained 7 diseases, of which "cancer of urinary tract" (DOID:3996) is the ancestral node of the other 6 diseases. However, the observed correlation between "germ cell cancer" (DOID:2994) and the largest module which has a size of 22 (Figure 4) doesn't show any direct link in the DO graph. Again, the network representation in Figure 4 provided additional insights to our analysis.

### Application on gene similarity

**Figure 5:**
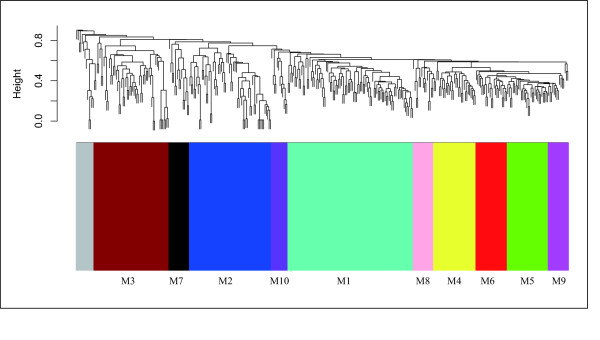
**Hierarchical clustering result of the obesity related genes**. The grey bar indicates the genes that could not be grouped into a certain module.

Here, by discussing the disease risk of obesity, we demonstrated another application of DOSim (using functions of calculating similarity between genes and DO-directed gene modules detection and annotation). Previous studies showed that obesity increased the risk of various diseases, such as type 2 diabetes, heart disease and certain types of cancer [30]. In this example, we used obesity related genes (651 genes) that were downloaded from the Phenopedia database[31]. Of the 651 genes, 361 had DO annotations. The similarities between these 361 genes were calculated using the BMA method on the Resnik measure (This is just one example. Users can choose to use any of the others in their applications). A gene similarity matrix *S *= [*s*_*ij*_]_361 × 361 _was constructed where *s*_*ij *_is the similarity between *i*th gene and *j*th gene in the gene set. After that an average linkage hierarchical clustering was performed and then a dynamic tree cutting method was applied (minimal module size is larger than 10) [24]. Finally, 10 different gene modules were obtained (Figure 5, Table 1).

**Table 1:** Gene modules of the obesity related genes

Cluster	Size	Average similarity	***p*-value**^**#**^	FDR^*^	**Representative GO annotation**^**§**^	**Representative KEGG annotation**^**§**^
M1	92	0.43	<1.0E-05	<1.0E-04	cholesterol homeostasis; high-density lipoprotein particle remodelling; triglyceride catabolic process	Insulin signaling pathway; Type II diabetes mellitus
M2	60	0.30	0.25	0.28	N/A^$^	Pyruvate metabolism; Galactose metabolism;
M3	55	0.30	0.29	0.29	feeding behavior; photoreceptor cell maintenance	Neuroactive ligand-receptor interaction; Circadian rhythm - mammal;
M4	31	0.50	<1.0E-05	<1.0E-04	response to estrogen stimulus; response to cytokine stimulus; cell aging	Pathways in cancer; Colorectal cancer; Endometrial cancer;
M5	30	0.62	<1.0E-05	<1.0E-04	response to lipopolysaccharide; response to glucocorticoid stimulus	Cytokine-cytokine receptor interaction; Toll-like receptor signaling pathway;
M6	23	0.55	<1.0E-05	<1.0E-04	positive regulation of phosphoinositide 3-kinase cascade; positive regulation of cholesterol esterification	Renin-angiotensin system; Prostate cancer
M7	15	0.34	0.12	0.16	N/A	Insulin signaling pathway
M8	15	0.43	6.0E-04	6.0E-03	blood coagulation; STAT protein nuclear translocation	Complement and coagulation cascades; Regulation of actin cytoskeleton
M9	15	0.53	<1.0E-05	<1.0E-04	response to interleukin-1; response to glucocorticoid stimulus	Hematopoietic cell lineage; Cytokine-cytokine receptor interaction
M10	12	0.40	1.5E-02	2.2E-02	N/A	N/A

# the original *p*-value calculated by permutation

* FDR using Benjamini and Hochberg multiple testing correlations

§ Refer to Additional file 3 for complete GO and KEGG annotations.

$ N/A indicates that there are no enriched GO or KEGG annotation for this module.

When the complete GO and KEGG annotations of these ten different gene modules were analysed (Additional file 3), we found different enriched biology functions and pathways for each module, indicating the complex pathogenesis of obesity. For example, the KEGG annotations of one of the clusters (M4) (Table 1) indicated that obesity is a factor that may lead to various cancers (e.g., colorectal cancer and endometrial cancer) and that obesity may also have a relationship with many signalling pathways (e.g., ErbB signalling pathway and Jak-STAT signalling pathway). However, the KEGG annotations of another cluster (M2) suggested that obesity may either affect the metabolism of many molecules or that the dysfunctional metabolism of these molecules may lead to the obesity (e.g., pyruvate metabolism and galactose metabolism). Similarly, the GO annotations of cluster M1 implied that obesity has a relationship with the biology process of cholesterol, lipoprotein and triglyceride (e.g., cholesterol homeostasis, reverse cholesterol transport, high-density lipoprotein particle remodelling and triglyceride catabolic process), while the GO annotations of cluster M3 suggested that obesity may be associated with eating habits (e.g., feeding behavior and drinking behavior). Both the GO and KEGG annotations of cluster M8 indicated that obesity is related to coagulation (blood coagulation in GO; complement and coagulation cascades in KEGG). These multilayered annotations successfully demonstrated the complex pathogenesis of obesity and suggested that the genes in the different gene modules would be potential drug targets for the corresponding diseases caused by obesity.

## Discussion

The DOSim package offers an easy and straight forward way to study disease similarity and gene similarity simultaneously in the DO. Additionally, other utilities implemented in the DOSim, such as function of gene module detection and gene module multilayered annotation, make better application of the DO and facilitate researchers. The presented two case studies highlight the usefulness of the DOSim in a real life scenario. We also provided the Additional file 4 which contains all the necessary R scripts to generate the above two case studies.

## Conclusions

The DOSim package advances the use of DO by integrating information theoretic similarity concepts for diseases and deriving disease similarity measures for genes in the powerful R system. Compared with the few existing bioinformatics tools for DO, e.g., FunDO, which explores disease information implied in the gene set by enrichment analysis, DOSim focuses on the computation of disease-disease and gene-gene similarities. Other utilities, such as function for gene module detection and gene module multilayered annotation, should help promote a better understanding of the complex pathogenesis of some disease risk phenotypes and the heterogeneity of some diseases. DOSim is available on the Comprehensive R Archive Network (CRAN) project or through http://bioinfo.hrbmu.edu.cn/dosim.

## Competing interests

The authors declare that they have no competing interests.

## Availability and requirements

**Project name**: DOSim

**Project home page**: http://bioinfo.hrbmu.edu.cn/dosim

**Operating system(s)**: platform independent

**Programming language**: R

**Other requirements**: none

**License**: GPL

## Authors' contributions

JL, BG, CW, FZ, SR and XL conceived the project and wrote the paper. JL, XC, CL and TL designed the software and performed the analyses. JL and BG designed the code and implemented the software. All authors read and approved the final manuscript.

## Supplementary Material

Additional file 1 **Pairwise similarity matrix between 128 cancer terms and a list of significant cancer pairs**. Similarities for these 128 cancers were computed by *getTermSim *function using the Wang measure. The threshold of similarity 0.43 was selected by permutation and the corresponding *p*-value was 0.01. The excel file contains three separate sheets named 'readme', 'similarity matrix' and 'significant disease pairs'. They contain the following information: Readme: Brief introduction to the file. Similarity matrix: Stores all the 180 cancers' pairwise similarities. Data coloured red are those with a similarity larger than 0.43, corresponding to *p*-value 0.01. Significant disease pairs: Represents the significant disease pairs at a significant *p-*value of 0.01 fetched from the 'similarity matrix'. Click here for file

Additional file 2 **The DO graph of the 128 cancer DO terms**. The DO graph of the 128 cancer DO terms was generated by "getDOGraph" function in the DOSim package. The 128 terms functioned as leaves, resulting in 378 terms in total. The 128 starting terms are represented as circles with different colours according to the modules they belong to. The additional 270 terms are represented as grey squares. Two modules coloured in brown and green are expanded as examples amd compared with the results in the Figure 3. Additionally, term DOID:2994 (germ cell cancer) is also expanded as an example and compared with the results in the Figure 4. Click here for file

Additional file 3 **Detailed annotation for ten obesity related gene modules **Ten modules of obesity genes were obtained by 'detectModule' function with minimal module size larger than 10 and using the 'tree' method. The module annotation was carried out by the R script in the Addtional file 4 (R_Code.R'). All GO and KEGG terms assigned to each module are at a significant level of FDR < = 0.01. Click here for file

Additional file 4 **R and Perl scripts used to generate the results in the two case studies **This zip file contains the 10 files, which were used to generate the results in the two case studies. Two files, the "R_Code.R" and the "get_significant_of_each_module.pl" are the main scripts that were used. A detailed description of all 10 files is available in the "Readme.txt" file. Click here for file
