# Supplementary figures and images for: DOSim: An R package for similarity between diseases based on Disease Ontology
Source: BMC Bioinformatics. 2011 Jun 29;12:266. doi: 10.1186/1471-2105-12-266 (PMC3150296; doi:10.1186/1471-2105-12-266)

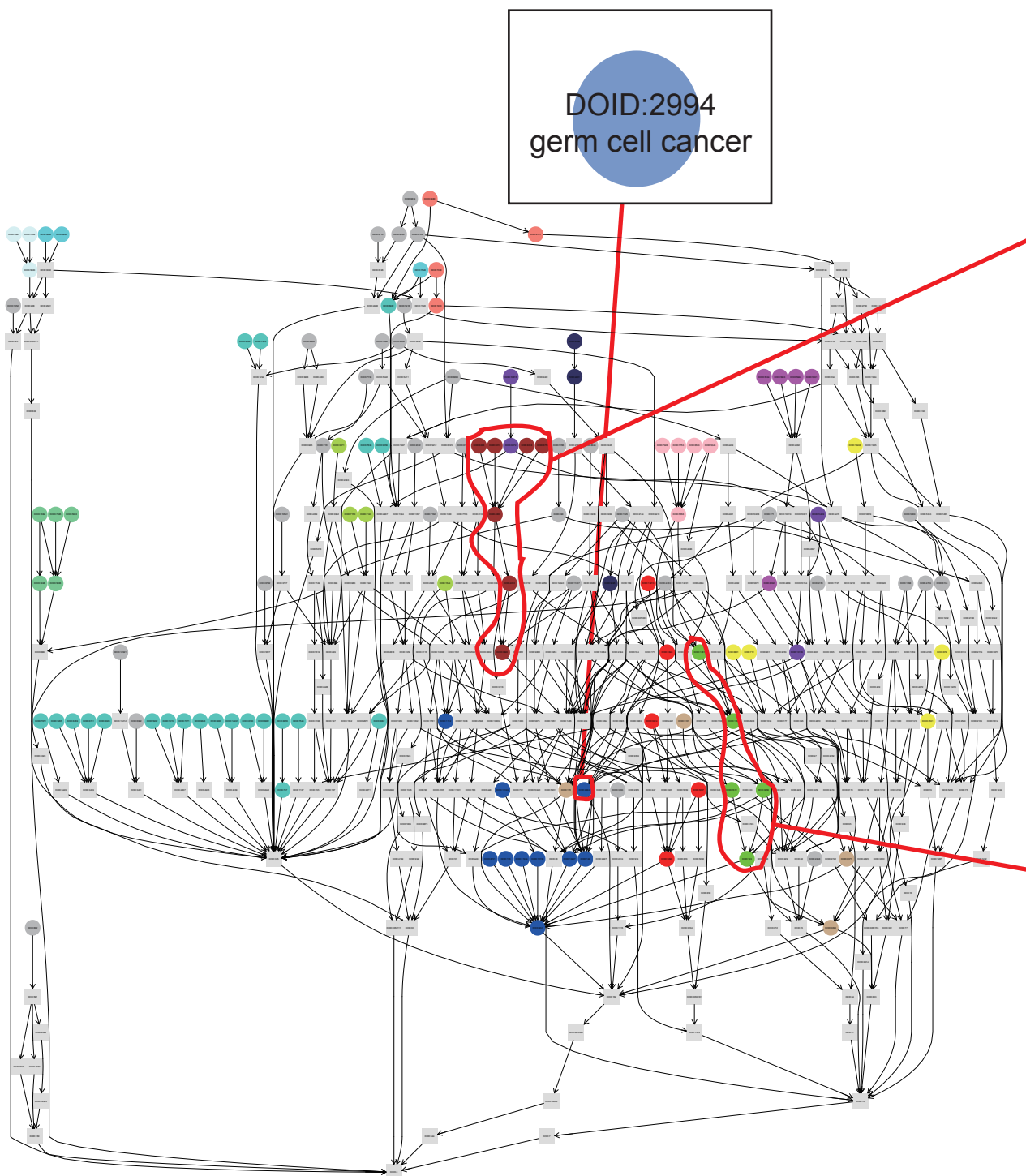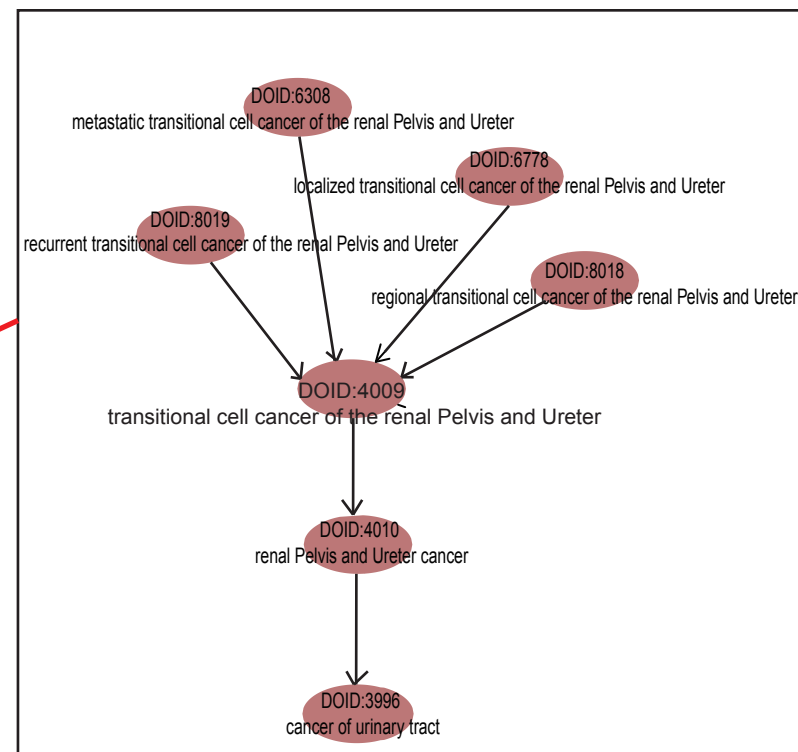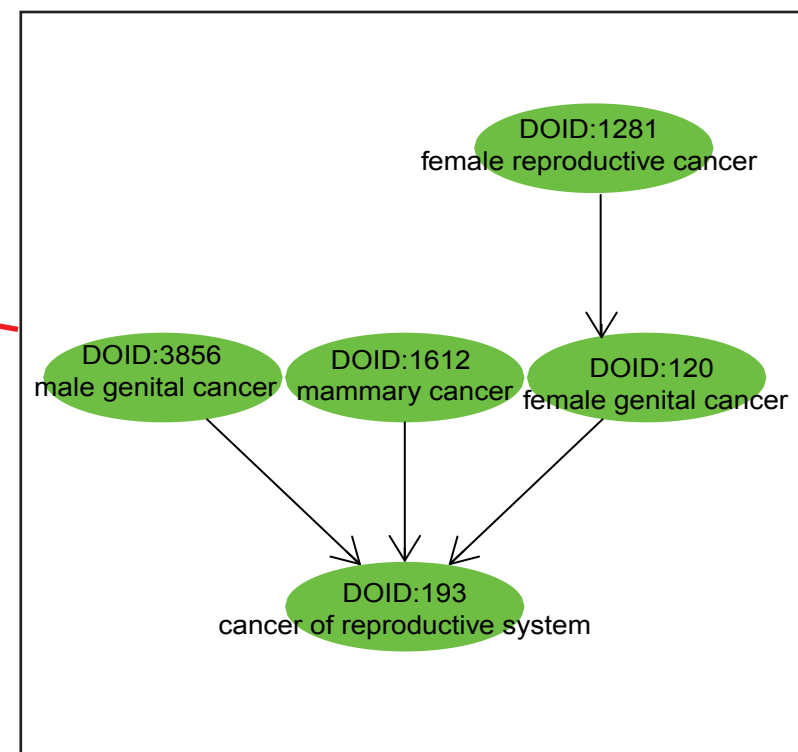

Supplement: Additional file 2 — The DO graph of the 128 cancer DO terms. The DO graph of the 128 cancer DO terms was generated by "getDOGraph" function in the DOSim package. The 128 terms functioned as leaves, resulting in 378 terms in total. The 128 starting terms are represented as circles with different colours according to the modules they belong to. The additional 270 terms are represented as grey squares. Two modules coloured in brown and green are expanded as examples amd compared with the results in the Figure 3. Additionally, term DOID:2994 (germ cell cancer) is also expanded as an example and compared with the results in the Figure 4. [file 1471-2105-12-266-S2.PDF]
